# Improvement of medical content in the curriculum of biomedical engineering based on assessment of students outcomes

**DOI:** 10.1186/s12909-017-0968-2

**Published:** 2017-08-04

**Authors:** Enas Abdulhay, Ruba Khnouf, Shireen Haddad, Areen Al-Bashir

**Affiliations:** Department of Biomedical Engineering, Faculty of Engineering, Jordan University of Science and Technology, Irbid, 22110 Jordan

**Keywords:** Students’ outcomes, Assessment, Biomedical, Medical, Curriculum, Improvement

## Abstract

**Background:**

Improvement of medical content in Biomedical Engineering curricula based on a qualitative assessment process or on a comparison with another high-standard program has been approached by a number of studies. However, the quantitative assessment tools have not been emphasized. The quantitative assessment tools can be more accurate and robust in cases of challenging multidisciplinary fields like that of Biomedical Engineering which includes biomedicine elements mixed with technology aspects. The major limitations of the previous research are the high dependence on surveys or pure qualitative approaches as well as the absence of strong focus on medical outcomes without implicit confusion with the technical ones. The proposed work presents the development and evaluation of an accurate/robust quantitative approach to the improvement of the medical content in the challenging multidisciplinary BME curriculum.

**Methods:**

The work presents quantitative assessment tools and subsequent improvement of curriculum medical content applied, as example for explanation, to the ABET (Accreditation Board for Engineering and Technology, USA) accredited biomedical engineering BME department at Jordan University of Science and Technology. The quantitative results of assessment of curriculum/course, capstone, exit exam, course assessment by student (CAS) as well as of surveys filled by alumni, seniors, employers and training supervisors were, first, mapped to the expected students’ outcomes related to the medical field (SOsM). The collected data were then analyzed and discussed to find curriculum weakness points by tracking shortcomings in every outcome degree of achievement. Finally, actions were taken to fill in the gaps of the curriculum. Actions were also mapped to the students’ medical outcomes (SOsM).

**Results:**

Weighted averages of obtained quantitative values, mapped to SOsM, indicated accurately the achievement levels of all outcomes as well as the necessary improvements to be performed in curriculum. Mapping the improvements to SOsM also helps in the assessment of the following cycle.

**Conclusion:**

The suggested assessment tools can be generalized and extended to any other BME department. Robust improvement of medical content in BME curriculum can subsequently be achieved.

## Background

The departments of Biomedical Engineering (BME) are usually founded to maintain pace with global developments in the fields of technology pertaining to healthcare. They are supposed to provide hospitals and health centers with qualified engineers in the areas of diagnosis, therapy, rehabilitation and research. The program offered at any department of biomedical engineering has to improve and to develop depending on the evolving inputs and local/global circumstances [1], especially those related to medicine and biology. However, many BM engineers suffer from the “easier said than done” work in medical fields or interaction with medical practitioners. The difficulty is mainly produced by the fragile medical content in many courses in BME [2]. Improvement of those courses is essential for complexity reduction. Furthermore, several calls are being proposed to develop, assess or improve programs that can attract students or medical practitioners/students who desire biomedical engineering based learning in medical context [3, 4] (e.g. pathology informatics, medical devices, genetic computation…etc.); The improvement of medical content in the curriculum should therefore be very carefully targeted. Consequently, in the process of continuous improvement in a BME department, measures should be taken to remedy the eventual shortcomings related to the medical and biological content in its courses and study plan (curriculum).

Traditionally, courses were defined in terms of their duration, syllabus and content (Content-based education). Clear statement as to what students were expected to learn was not on the agenda [5]. On the other hand, Outcome-Based Education (OBE) is the approach where decisions about the curriculum are driven by the outcomes the students should display by the end of the course [6]. It provides an explicit statement of what the curriculum is setting out to achieve [7]. The transfer of education system from the traditional approach to Outcome Based Education (OBE) had given a significant change in many educational institutions worldwide [8]. The main elements/phases of OBE are: defining learning outcomes, outcome-based development of curriculum, outcome-based curriculum assessment and outcome-based continuous improvement. The implementation of OBE implies the interaction between all stakeholders: students, instructors, faculty, educational environment, course/curriculum planning/assessment committees and advisory board.

In the first phase, outcomes should encompass skills, abilities, and knowledge that the students should attain by the time of their graduation. Several interrelated dimensions should be considered. The key characteristics for the identification of medical learning outcomes have been established by a number of studies. In [9], a three-circle outcome model has been adopted: outcomes related to the performance of tasks expected, outcomes related to the approach adopted by the doctor to the performance of tasks, and outcomes related to professionalism. The design down process for development of outcomes has been summarized as: (1) generation of exit outcomes, (2) phase outcomes, (3) course outcomes and finally (4) lesson outcomes.

In the second phase, Bloom’s Taxonomy is one of the important approaches for designing educational learning processes [10]. It comprises aspects related to creating, evaluating, analyzing, applying, understanding and remembering.

In the third phase, OBE is more than a traditional method that is only based on GPA and course completion. It has been indicated by [11] that the correct type of Learning Outcomes Assessment for higher education should integrate several components that are instructor-based and student-based to give an accurate picture of attainment of the learning outcomes. The New World Kirkpatrick Model is the worldwide standard for evaluating the effectiveness of teaching. It considers the value of teaching across four levels: reaction, learning, behavior and results [12]. Reaction level is the degree to which students find the teaching favorable, engaging and relevant. Learning level is the degree to which students acquire the intended knowledge, skills, attitude, confidence and commitment. The third level is the degree to which students apply what they learned. The last level is the degree to which short-term observations and measurements suggest that critical behaviors are on track to create a positive impact on desired results [12]. Direct measures of assessment are measures in which the products of student work are evaluated (ex. activities from coursework) while indirect measures of assessment are those in which students judge their own ability to achieve the learning outcomes [13]. Qualitative assessment involves open-ended interviews and questions [14], observations (notes, checklists, rubric.etc.) [15], records and documents [14]. However, whenever criteria are used with a qualitative method, the process of inductive discovery is diminished [16]. On the other hand, the quantitative approach comprises structured interviews, questionnaires [17], and tests. It can be generalized and customized more easily than qualitative methods. The most used tools are student, alumni and employer surveys, performance assessment, rubrics, portfolios, general knowledge and skill measures [18]. Surveys are easily administered but do not guarantee direct evidence of student learning. The most authentic tool is the external performance assessment. However, it is not easy to implement.

In the fourth phase, a failure to achieve the agreed outcomes almost certainly identifies a problem with the curriculum [19]: good curriculum means good study progress. Improvement of the curriculum should be based on the assessment results and eventual modifications of outcomes based on local/global circumstances.

As will be detailed later in the discussion section, improvement of medical content in BME curriculum based on qualitative assessment processes, comparison with high-standard programs or implicit inter-relation between the medical content and the BME expected technical outcomes has been approached by a number of studies [3, 4, 13, 19–33]. However, the major limitations of the previous research are the high dependence on surveys or pure qualitative approaches as well as the absence of strong focus on medical outcomes without implicit confusion with the technical ones. The proposed work presents the development and evaluation of an accurate/robust quantitative approach to the improvement of the medical content in the challenging multidisciplinary BME curriculum which includes mixed technology and biomedicine elements.

## Methods

The main goals of the present work are: (1) the clear and accurate identification of student medical outcomes SOsM (satisfying the requirements of Outcome Based Education) without confusion with technical outcomes, (2) the separate quantitative assessment of every medical outcome & the robust quantitative assessment of every medical outcome by multiple quantitative assessment formats (different direct and indirect tools) that complement each other and increase accuracy, (3) the precise targeted evaluation/improvement of curriculum medical content in light of quantitative assessment results, and finally (4) the implementation intended for more explanation about the approach evaluation. The present work takes the BME Department at Jordan University of Sciences and Technology (JUST) as an example to which the methodology of the paper is applied. The department is the first of its kind in Jordan. The BME program at JUST was granted accreditation by the Engineering Accreditation Commission EAC (USA) of the Accreditation Board for Engineering and Technology (ABET) effective 2007. Renewal of accreditation was granted as well in 2016 [2]. ABET is a non-profit and non-governmental organization, in the United States of America, that accredits university programs in the fields of applied science, computing, engineering and engineering technology based on high graduates quality and department standards [34, 35]. The mentioned department adopts a plan targeting particularly the aspects related to the assessment of the BME mission statement, program educational objectives (PEOs) and student outcomes (SOs) [34] by maintaining the BME program constituencies-feedback regular analysis. The process starts by defining the BME program educational objectives (PEOs) from which emerge student outcomes (SOs) [36], since outcomes are the most important part of the educational process. Also, sub-outcomes are defined for each course in the program, based on the material in each course, and are linked to the students’ outcomes. In the implementation section, only the elements of assessment/improvement interesting to international readers will be introduced without presentation of the special details related to the BME department at JUST University.

### Identification of student outcomes related to biomedicine (SOsM)

#### Program constituencies

The significant primary constituents of the program are:

- Faculty members of the BME program: The faculty members strongly contribute to the advancement of their department. They are actively engaged in teaching and research activities in the whole spectrum of BME.
- Employers of BME graduates: The employers of the BME program graduates include institutions in both public and private sectors.
- Advisory board: Provides feedback to the department that helps forging the program’s policies and objectives. The board comprises of members, doctors, directors and managers from several health and biomedical Institutions. This board is regarded as the most important primary constituent of formulating and adjusting the program.
- Alumni of the BME program: The BME department pumps biomedical engineers into the market. The graduates cover the local market, can have recorded international presence, and some of them pursue advanced degrees in recognized graduate schools around the globe.
- Student training supervisors: These are experienced individuals assigned by their employers in the institutions where BME students get their required practical training prior to graduation.
- Undergraduate BME students: Make the main body and focus of the department. They are the students currently enrolled on a full-time basis.

#### Engagement in interrelated dimensions

One of the most important raised issues during the process of establishing the goals of the BME program at the department was the conviction that students get engaged in several interrelated dimensions that have substantial impact on forming students’ characters for their future careers. The dimensions are categorized as follows: D1. Scholarship and knowledge, D2. Intellectual communication, D3. Community building, D4. Leadership, and D5. Spirituality and values. These dimensions stem mainly from the principles that underline the adult learning process. BME Students are engaged in the interrelated dimensions contributing to the development of the whole biomedical engineer as a person. In D1, the goal is that the students become distinguished scholars in their field of choice, be dedicated to advancement of knowledge, and prepared for higher studies and fruitful careers as well as lifelong learning. The indicators relevant to this dimension include: being prepared for higher study, being prepared for a future career, demonstrating substantial general knowledge and coping well with the core field of study. In D2, the qualities of intellectual communication are necessary for student effective learning, clear idea expression, and successful application of knowledge acquired to new situations. The indicators relevant to this dimension are: utilizing modern methods and tools, adopting and applying resources, demonstrating the skills of communication (oral and in-writing), critical thinking (synthesis, evaluation, analysis, integration, and application) and problem-solving. In D3, this dimension intends to build an inclusive community by working on welcoming collaboration with others regardless of their ethnic group, faith, or gender. An inclusive community leads to building noble values of respect and compassion to human life and dignity. Indicators relevant to this dimension include: working collaboratively with others, and demonstrating acceptance of others with respect to their differences. In D4, students are motivated and encouraged to take the initiative to get engaged voluntarily in local and global issues of significance. Indicators relevant to this dimension include: demonstration of an understanding of the interconnectedness of global and local concerns, and recognition of contemporary issues. In D5, this dimension aims at helping students determine the set of principles that guide their actions and define their relationships with others. Indicators relevant to this dimension include: defining and articulating one’s own values and beliefs, and making informed ethical decisions in personal and professional situations.

#### Program educational objectives (PEOs)

The process starts by defining the BME program objectives from which emerge student outcomes (described in the next section), since outcomes are the most important part of the educational process. The program educational objectives should encompass the student outcomes and be consistent with university, faculty, department and program missions as well as with the above inter-related dimensions. All the vision and mission statements as well as PEOs are published in JUST website [37].

In the beginning of the educational process, the Program Educational Objectives are identified to generally provide high-quality education, research, and service. The objectives are consistent with EC2000 [36]. Following many department committees and Advisory Board meetings, the BME department established a set of Program Educational Objectives that focus on the expected accomplishments of the students in their careers. Afterwards, extensive meetings -overseen by a departmental committee and involving relevant parties including the student body, public healthcare institutions, private sector representatives, department alumni, and potential employers- preceded writing down the objectives in their final form and resulted in significant feedback from all those entities. The result of this process was the following Program Educational Objectives:

PEO1.Visionary engineers and problem solvers, utilizing a breadth of scientific knowledge to address contemporary issues at the interface of engineering, medicine, and biology within a global, societal, and economic context.

PEO2. Leaders in biotechnology and medical industries both in the public and private sector capable of serving national and regional industries, hospitals, and government agencies.

PEO3. Ethically and socially conscious professional biomedical engineers functioning well in multi-disciplinary teams, effective in communicating ideas and technical information.

PEO4. Independent learners who can master new knowledge and technologies, as well as, successfully engage in post-graduate studies and scientific research in engineering, medicine and biomedical sciences.

#### Student Outcomes (SOs), Student Outcomes Related to Biomedicine (SOsM) and courses sub-outcomes

Student Outcomes (SOs) were developed with participation of the BME program primary constituencies at the same time as the Program Educational Objectives. The SOs list includes the medical SOsM and the technical outcomes. The different committees believed that in order to establish the SOs that can ensure the achievement of the PEOs, students should be engaged in the five interrelated dimensions. The expected students’ outcomes related to biomedicine (SOsM) have therefore been determined as follows:

1. The ability to function within multi-disciplinary teams including physicians and medical practitioners (dimensions: D2, D3 and D5).
2. Graduates must demonstrate adequate knowledge of physiology, anatomy, biology, and the capability of applying acquired skills to solve the problems specifically at the interface of medicine/biology and engineering (dimensions: D1, D3 and D4).
3. Graduates must demonstrate an ability to make measurements on, and interpret data from, living systems, addressing the problems associated with the medical and biological interaction between living and non-living materials and systems (dimensions: D1 and D3).

The BME program primary constituencies felt the Student Outcomes (SOs) should directly support the Educational Objectives. The medical outcomes (SOsM) have therefore been determined in consistency with the program education objectives (PEOs), as illustrated in Table 1. The table shows a strong correlation between any given PEO and at least one of the SOsM. During the process of establishing the SOs, the following were also taken into consideration: the national, regional and global needs; ABET criteria; university, faculty of engineering, and BME department strategic plans; the feedback from health and biomedical institutions, alumni and advisory board through meetings; and the comments of students through interviews and other contacts.

**Table 1:** Relationship of BME SOsM to Program Educational Objectives

	BME student medical outcomes		
BME PEOs	1	2	3
PEO1	□	■	■
PEO2	□	◘	■
PEO3	■		
PEO4	□	■	■

The solid square presents the strongest correlation between a PEO and an SOM; the half square indicates a moderate relationship between a PEO and an SOM; the lowest level of correlation is presented by an empty square

The list of medical outcomes indicates that the items address all the fundamental medical skills, abilities, and knowledge the BME graduate is expected to acquire by the time of graduation.

Courses sub-outcomes are defined based on the material in each course and Bloom’s Taxonomy requirements. They are linked to SOsM. Examples of sub-outcomes are:

- Outcome 1: to plan/organize/distribute tasks in a team work environment, participate effectively in multi-disciplinary teams, handle a crisis situation using teamwork, and follow up team progress.
- Outcome 2: to recognize the impact of biology, physiology and biotechnology in biomedical engineering and apply the concepts to solve problems.
- Outcome 3: to use the acquired knowledge to simulate real life situations, to recognize the interaction between living and non-living systems, to conduct experiments, and to use laboratory equipment, material and procedures in a safe manner.

Technical outcomes are also listed below with their related dimensions and PEOs:

- The ability to apply knowledge of mathematics, science and engineering. (D1,D2/PEO1,2)
- The ability to design and conduct experiments, as well as to analyze and interpret data. (D1,D2/PEO1,2)
- The ability to design a system, its components or processes to meet the desired needs (D2,D3/PEO1,2)
- The ability to identify, formulate, and solve engineering problems. (D1,D2/PEO1,2)
- An understanding of professional and ethical responsibilities. (D5/PEO2,4)
- The ability to communicate effectively. (D2/PEO3,4)
- The broad education necessary for understanding the impact of engineering solutions in a global and societal context. (D4/PEO1–4)
- Graduates must recognize the need for, and the ability to engage in life-long learning (D1,D2,D4/PEO1–4)
- Graduates must have knowledge of contemporary issues. (D4/PEO4)
- The ability to use the techniques, skills, and modern engineering tools necessary for engineering practices. (D1,D2/PEO1,3–4)

Examples of sub-outcomes related to two selected technical outcomes are presented below:

- Outcome: *To demonstrate that graduates have an ability to identify, formulate, and solve engineering problems*. Sub-outcomes: To build upon the learned theories to address new areas of Biomedical Engineering, develop appropriate strategies for identifying and solving engineering problems, make appropriate assumptions to enable reaching a practical solution as well as to assess the validity of the solution and how it is impacted by the assumptions.
- Outcome: *To demonstrate that graduates have an ability to design a system, component, or process to meet desired needs.* Sub-outcomes: to analyze and synthesize biomedical engineering operations including integrated complex systems consisting of multiple processes, design biomedical engineering processes/instrumentation and their components/units to meet realistic technical/safety/economical/environmental/social/ethical constraints and to apply modern computer tools/packages to process design and analysis.

### Separate quantitative assessment of every medical outcome by multiple quantitative assessment formats

If students can demonstrate achievement of the medical and technical outcomes by the time of their graduation then the graduates are prepared to attain the stated Program Educational Objectives a few years after graduation. The process of quantitative evaluation of achievement degree of students’ medical outcomes (SOsM) involves eight assessment tools. They are divided into direct and indirect tools as follows:

- Direct tools: curriculum/course assessment, exit exam and capstone course (graduation project)
- Indirect tools: training survey, students exit survey, students’ assessment of course (CAS), alumni survey and employer survey.

All surveys have been structured in a way consistent with the previously mentioned medical outcomes. Their items have then been mapped to SOsM. The assessment process has been approved by J.U.S.T Students Surveys/ABET Ethics Committee. The surveys are made available to program constituencies and interested persons via [2, 38]. In addition, several meetings were organized to explain to all program constituencies the process and the objective of assessment as well as the possible publishing of results.

The assessment results are aggregated to evaluate the individual outcomes (SOsM) separately on a scale of 1 to 5. The present work has established the following success criteria for each outcome:

SI: Suggested Improvement. The students’ outcome has been met, but recommendations for some improvement may be suggested. The score is between 3.0 and 5.0.

NI: Needs Improvement. The students’ outcome has been marginally met. Improvement should be suggested and implemented. The score is between 2.5 and 3.00.

MI: Major Improvement. The students’ outcome has not been met. Major improvement should be suggested and implemented. The score is less than 2.5.

The following sub-sections discuss in detail the methods for the suggested assessment tools.

#### Course and Curriculum Assessments & Assessment by students (1st and 2nd assessment tools)

The curriculum assessment process accumulates the individual contributions from all courses in the BME program to the students’ medical outcomes in order to assess the contribution of the entire curriculum. The following sections describe the process in detail.

##### Assessment of course outcomes *&* student performance (course level)

The process starts by developing the course syllabus. In developing a course syllabus, the course general outcomes are set first from the SOsM list. These outcomes for each course define the expected particular sub-outcomes. These may drastically differ from one course to another. This is the case because of the particular nature of each course. Once each course outcomes and sub-outcomes have been derived, the course is designed and delivered. Course assessment tools are then used to collect data indicating whether the course material has been properly delivered to the students. The proper delivery is indicated by the quantitative answers to the following questions: “to what extent should the course cover the outcome?”, “to what extent did the course actually cover the outcome by related activities?”, “to what extent did the course cover the outcome, from students’ point of view?” and “to what extent did the students achieve the expected outcomes?”. The answers are found out using the indices presented in the next sub-sections:

*“To what extent should the course address the outcome?” (Part 1).*

Assessment of the Course by the Faculty (CAF): Each faculty evaluates each of the course intended outcomes 1, 2 and 3 on a scale 1–5. It is decided by the faculty member, teaching the course, and approved by the focus group and the BME department council. The evaluation corresponds to the extent the faculty feels the class should help the students achieve. Table 2 illustrates an example related to the course “Physiological modeling”. For example, if team work is not essential in the course (e.g. to be only considered in a mini project), it should be assigned a smaller weight compared to outcome 2 because the content focuses on anatomical/physiological concepts. Outcome 3 has smaller weight than outcome 2 as the interaction between human body and instrumentation is rather highlighted by other courses in the curriculum. The technical outcomes have the overall weight 5. The weight for every single technical outcome will be detailed in the next sub-section. The attribution of high weight to the technical outcomes is due to the fact that the course emphasizes several technical aspects: application of differential equations, analysis and validation of simulated data, design of a control system with its components, and the use of the engineering techniques in modeling issues. The scale 1–5 can be transformed to a percentage % scale by dividing the weight by the total. For example, the value 1 related to the first medical outcome can be transformed to 1/(5 + 1 + 3 + 1) = 10%.

**Table 2:** Assessment by faculty of the course physiological modeling

Student outcomes	Technical outcomes	Medical outcome 1	Medical outcome 2	Medical outcome 3
Assessment of the Course by the Faculty	5	1	3	1

The scale 1–5 can be transformed to a percentage value by dividing the weight by the total. For example, the value 1 related to the first medical outcome can be transformed to 1/(5 + 1 + 3 + 1) = 10%

*“To what extent did the course address the outcome (from students’ point of view)?”*

Assessment of the Course by Student (CAS): Student assessments are surveys filled out at the end of each semester. These student assessments are intended to provide the students’ views of their opportunities to master the students’ outcomes. Not every class is expected to impact all students’ outcomes. To minimize difficulty in filling the survey, the survey translates each course outcome, as presented in the course syllabus, into examples (course and lesson sub-outcomes) that are emphasized during teaching the course. The survey asks students to evaluate each course outcome based on a 1–5 scale, where 1 is poor and 5 is excellent. The bases of evaluation should be on student’s feeling of how the course has helped him/her achieve the abilities, attributes, and skills as described in the outcomes. Table 3 illustrates an example about five sub-outcomes among the intended sub-outcomes of the course “Physiological modeling”. The first course sub-outcome is related to the student medical outcome 1. The two following course sub-outcomes are related to the student medical outcome 2, while the last two course outcomes are related to the student medical outcome 3. For every student, the final value of an outcome is the average of the related sub-outcomes values.

**Table 3:**
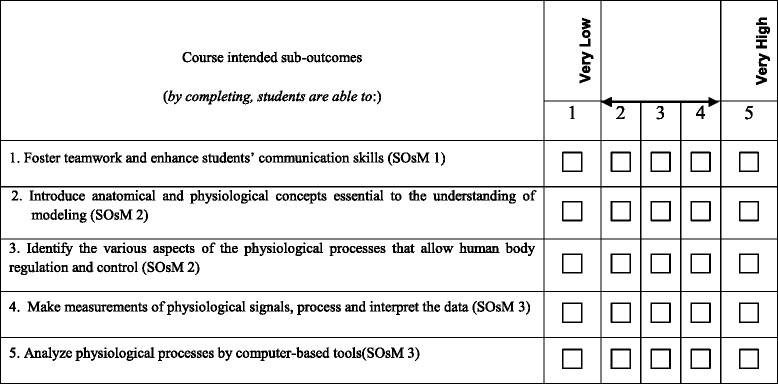
A part of the form of course assessment by students to be filled out for the course ‘Physiological modeling’

**Fig. 1:**
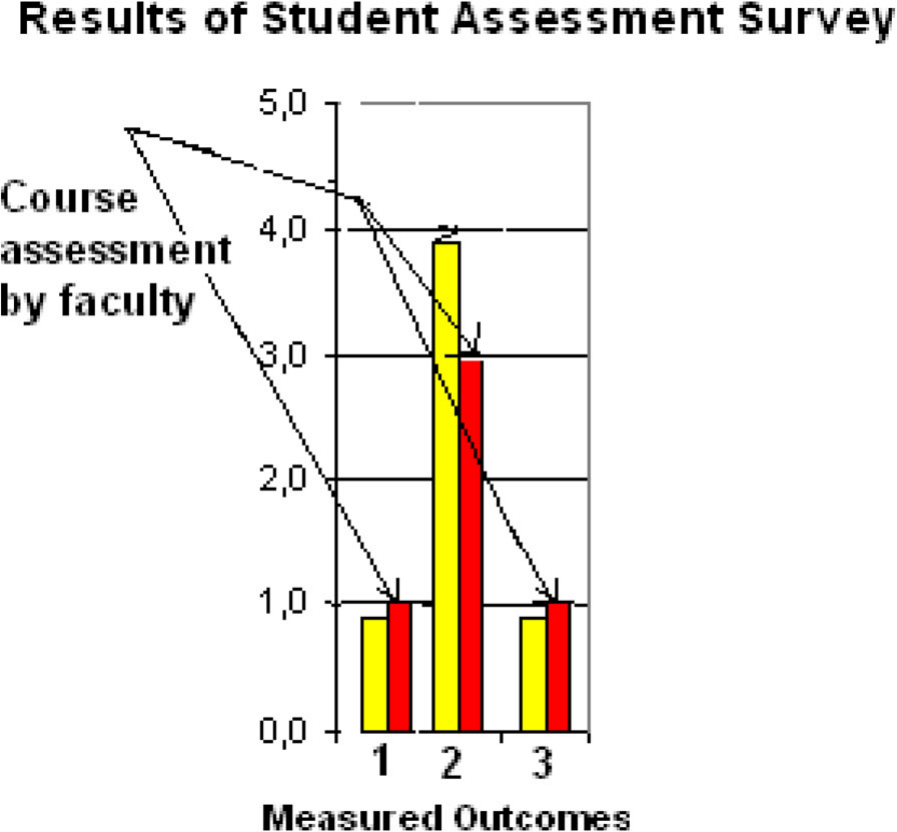
Example of analyzed CAS results (‘Physiological modeling’ course)

Figure 1 illustrates an example of comparison between the obtained assessments by students and by faculty for the course ‘Physiological modeling’.

The quantitative threshold useful for the evaluation/improvement of the course and applied to the value of the difference between CAF and CAS will be introduced in the section of targeted improvement.

*“To what extent should the course address the outcome?” (Part 2).*

The expected *Target* percentage of addressing a medical outcome in a particular course is the CAF value, explained earlier, transformed to a percentage value. Addressing all outcomes is considered as 100%. Then, the *Target* values are fed into CAP (Course Assessment Program). CAP is a developed Excel program that breaks down each course’s content in terms of criteria (outcomes) [2, 39, 40]. Table 4 illustrates an example of *Target* determination for the course “Physiological modeling”. Every separate medical or technical outcome is assigned a *Target* value. The medical outcome 2 has the highest weight since the course focuses on many aspects of physiology, physiological models and engineering/medicine interface. The medical outcome 1 is assigned 10%. In the syllabus, outcome 1 is to be considered in homework where students should interact, with medical doctors, concerning the significance of the results of different pathologies simulation. The medical outcome 3 is assigned 10%. In the syllabus, outcome 3 is to be considered in computer-based homework where aspects of interaction between respiratory system and ventilator are studied and analyzed. The technical outcomes 3 and 10 have high weights because the course focuses on the design of control systems with static and dynamic properties and of mass transport processes as well as on the application of electrical and mechanical techniques to the several forms of simulation. On the other hand, technical outcomes 5, 7 and 8 got zero weights because the course does not target ethical, societal or life-long learning aspects. Moderate weights have been assigned to the technical outcomes 1, 2, 4, 6 and 9 because the course approaches moderately the experimental physiology, solving engineering problems, written and oral communication skills, and analysis of contemporary scientific articles about modeling.

**Table 4:** An example of *Target* percentages for SOsM in the course ‘Physiological modeling’

The Instructor	Dr. Enas Abdulhay
The course (Name)	Physiological modeling
The course (code)	BME 531
The number of credit hours	3
Outcome	Target
Technical outcome 1	5
Technical outcome 2	2
Technical outcome 3	20
Technical outcome 4	5
Technical outcome 5	0
Technical outcome 6	3
Technical outcome 7	0
Technical outcome 8	0
Technical outcome 9	2
Technical outcome 10	13
Medical outcome 1	10
Medical outcome 2	30
Medical outcome 3	10
Total	100%

The medical outcome 2 has the highest weight since the course focuses on many aspects of physiology and physiological models. The medical outcome 1 is assigned 10% because it is to be considered only in homework where students should discuss, with medical doctors, the significance of the results of different pathologies simulation. The medical outcome 3 is assigned 10% because it is to be considered only in computer-based homework where interaction between respiratory system and ventilator is studied

*“To what extent did the course address the outcome via related activities?”:*

It is essential that the instructor provides the relative percentages of the assessment activities in the course. The percentages are the marks assigned to every activity given that the course total mark is 100. Table 5 illustrates an example related to the course “Physiological modeling”.

**Table 5:** Example of mark percentage distribution over different assessment activities in the course ‘Physiological modeling’

Activity	Mark
First Exam	25
Second Exam	25
Final Exam	40
Quiz1	2
Quiz2	2
HW1	2
HW2	2
Project	2
Total mark	100

The instructor is then advised to map every question in every assessment activity (or every assessment activity as one block) to SOsM. As every question addresses the medical outcomes by different proportions, the instructor should determine the percentage weight of every outcome in every question, given that the total percentage of all outcomes in the question is 100%. Table 6 illustrates an example of outcomes percentage values in the questions of final exam activity. Table 7 illustrates the corresponding calculated marks. The mark of every outcome in every question is calculated by the Excel program as: (mark of question × percentage of outcome in the question).

**Table 6:** Percentage of every outcome in every question in the final exam of the course ‘Physiological modeling’

Final exam															
		Technical outcomes (%)										Medical outcomes (%)			Total (%)
Part	Mark	1	2	3	4	5	6	7	8	9	10	1	2	3	
Part a.	10	3	6	11	0	0	0	0	0	0	0	5	70	5	100%
Part b.	5	5	0	0	10	0	10	0	0	30	0	0	40	5	100%
Part c.	5	0	0	0	0	0	5	0	0	5	80	0	5	5	100%
Part d.	10	5	5	20	5	0	5	0	0	0	50	0	5	5	100%
Part e.	10	0	0	20	10	0	5	0	0	2	50	0	7	6	100%
Total	40

**Table 7:** Outcomes mark distribution over the questions of final exam in the course ‘Physiological modeling’

		Marks of medical outcomes		
Part	Max. Mark	1	2	3
Part a.	10	0.5	7	0.5
Part b.	5	0	2	0.25
Part c.	5	0	0.25	0.25
Part d.	10	0	0.5	0.5
Part e.	10	0	0.7	0.6
Sum		0.5	10.45	2.1

The mark of every outcome in every question is: (mark of question × percentage of outcome in the question)

The course assessment Excel program checks then if the instructor was successful in using the *Tools* (assessing the student performance by the course activities such as exams, HWs, quizzes, projects …etc.) with the same weights as the intended *Target* for course outcomes. The *Tool* value for an outcome is calculated via the Excel program by the summation of the marks attributed to that outcome in all course activities. Table 8 illustrates an example. The course activities considered in Table 8 in *Tools* are all those mentioned in Table 5. The contribution of every assessment activity to *Tools* is calculated by the same procedure implemented above for final exam. Note that *Tool* values can be considered as percentages because the overall mark of the course is 100.

**Table 8:** *Target* and *Tool* values for the course ‘Physiological modeling’

Medical outcomes	1	2	3
‘Target’	10	30	10
Contribution of final exam to ‘Tools’	0.5	10.45	2.1
‘Tools’	8	33	9

The ‘*Tool*’ value for an outcome is calculated through the program by the summation of the marks attributed to that outcome (in all assessment activities). The assessment activities considered herein are those mentioned in Table 5. The contribution of every assessment activity is calculated by the same procedure as for final exam discussed above

“*To what extent did the students achieve the expected outcomes?”*

The course assessment program checks also if the class has satisfactorily passed each outcome criterion. A passing mark would be achieved when at least 60% of students (or overall average) score 60% mark or better for a given outcome based on *Tools* used. The interesting point is that a class scoring very high may be considered as unsuccessful if the individual outcomes are not met. Tables 9 and 10 illustrate examples of student marks in their final exam and their corresponding *Score* mapped to SOsM, respectively. The mapped *Score* in every question is calculated based on the student mark in that question and the percentage of the outcome in that question. The program is hence also capable of performing the individual analysis of every student’s *Score* for every outcome.

**Table 9:** Students’ marks of the different questions of final exam in the course ‘Physiological modeling’

Final exam					
Part	Maximum mark	Marks of students			
		Student 1	Student 2	Student 3	Student 4
Part a.	10	6	10	2	10
Part b.	5	2	5	1	4
Part c.	5	1	0	1	4
Part d.	10	10	8	9	9
Part e.	10	3	8	5	7

**Table 10:** Students’ marks of the different questions and outcomes in the final exam of the course ‘Physiological modeling’

Final exam				
Medical outcomes		1	2	3
Student 1	Part a.	0.3	4.2	0.3
	Part b.	0	0.8	0.1
	Part c.	0	0.05	0.05
	Part d.	0	0.5	0.5
	Part e.	0	0.21	0.18
Student 2	Part a.	0.5	7	0.5
	Part b.	0	2	0.25
	Part c.	0	0	0
	Part d.	0	0.4	0.4
	Part e.	0	0.56	0.48
Student 3	Part a.	0.1	1.4	0.1
	Part b.	0	0.4	0.05
	Part c.	0	0.05	0.05
	Part d.	0	0.45	0.45
	Part e.	0	0.35	0.3
Student 4	Part a.	0.5	7	0.5
	Part b.	0	1.6	0.2
	Part c.	0	0.2	0.2
	Part d.	0	0.45	0.45
	Part e.	0	0.49	0.42
AVG (sum of marks for every student)		0.35	7.03	1.37

The mapped *Score* in every question is calculated based on the student mark in that question and the percentage of the outcome in that question

Table 11 illustrates the comparison between the ‘*Target*’, ‘*Tools*’ and ‘*Score*’. *Target* is the distribution of the weights of outcomes determined by the faculty member. *Tool* is the extent of student assessment activities according to the outcomes. *Score* is actually what the students have achieved in the course activities according to the outcomes. The contribution of final exam to ‘*Score*’ for a particular outcome is the average of all related scores achieved by all students as illustrated in Table 10. The contribution of the other activities to *Score* value can be calculated by the same procedure implemented for the final exam. The overall value of *Score* is the sum of contributions from all assessment activities. Note that *Score* values can be considered as percentages because the overall mark of the course is 100.

**Table 11:** *Target*, *Tool* and *Score* values for the course ‘Physiological modeling’

Medical outcomes	1	2	3
‘Target’	10	30	10
Contribution of final exam to ‘Tools’	0.5	10.45	2.1
Contribution of final exam to ‘Score’	0.35	7.03	1.37
‘Tools’	8	33	9
‘Score’	6	24	6

*Score* is actually what the student has achieved in the course activities. The contribution of final exam to ‘*Score*’ for a particular outcome is the average of all related scores achieved by all students in the previous table. The contribution of the other activities to *Score* value can be calculated in the same way as implemented for final exam discussed above. The overall value of *Score* is the sum of contributions from all assessment activities. Note that *Score* values can be considered as percentages because the overall mark of the course is 100

*Comparison between the quantitative answers to the four raised questions:*

The comparison between the values of (CAF, CAS), (*Target*, *Tool)*, and (*Tool*, *Score)* should then be studied and discussed by the focus groups, at the end of the semester, for course evaluation. (CAF, CAS) comparison indicates the difference between “the extent (scale 1–5) to which the course should address the outcomes from instructor point of view” and “the extent (scale 1–5) to which the course has addressed the outcomes from students point of view”. (*Target*, *Tool)* comparison indicates the difference between “the extent (percentage) to which the course should address the outcomes, from instructor point of view” and “the extent (percentage) to which the course activities have indeed addressed the outcomes’. (*Tool*, *Score)* comparison indicates the difference between ‘the extent (percentage) to which the course activities have indeed addressed the outcomes’ and ‘the extent (percentage) to which the course activities, addressing the outcomes, have been successfully performed/solved by students”. The quantitative thresholds useful for the evaluation/improvement of the course and applied to the values of the previous mentioned differences will be introduced in the section of targeted improvement. The course assessment program shows warning messages when the comparison indicates educational problems (based on thresholds). The focus groups summarize the course assessment results in a report and submit it to the curriculum committee which, in turn, discusses it and submits the recommendations to the department council.

##### Assessment of curriculum outcomes *& student performance* (curriculum level)

The curriculum assessment process accumulates the individual contributions from all courses in the biomedical Engineering program to the student outcomes in order to assess the contribution of the entire curriculum. The evaluation of the entire curriculum is indicated by the answers to the following questions: “to what extent should the curriculum cover the outcome?”, “to what extent did the curriculum actually cover the outcome by related activities?”, “to what extent did the curriculum actually cover the outcome, from students’ point of view?” and “to what extent did the students achieve the covered outcomes?”.

The results from CAP of each course are fed into DAP (Department Assessment Program, which refers to assessment of curriculum outcomes). It is a developed Excel program that adds up all outcomes measures in all courses in the department designated as *Target*, *Tool* (student assessment components) and *Score* (student performance relevant to Tools used) [2, 39]. Thus, the overall picture of the curriculum is produced in terms of number of credit hours the department spends in each criterion (outcome). Each course has a “seat” in the DAP program. e.g. if *Target* = 33.33% for outcome 1 in a 3 credit-hour course X, the target number of credit hours (seat) of outcome 1 related to that course in DAP is 1 credit hour. If the total number of curriculum credit hours is, for example, 50 then the contribution of the course X to the entire curriculum Target of outcome 1 is (1/50). Table 12 illustrates a numerical example of curriculum Target, Tool and Score. The quantitative thresholds useful for the evaluation/improvement of the curriculum and applied to the difference values between Target, Tool and Score will be introduced in the section of targeted improvement. The quantitative answer to the question “to what extent did the curriculum actually cover the outcome, from students’ point of view?” will be explained in the sub-section D.

**Table 12:** Calculation of *Target*, *Tool* and *Score* values for outcome 1 in a curriculum consisting of four courses

Outcome 1							
Course	Credit hours CH	Target	Tool	Score	Target CH of outcome	Tool CH of outcome	Score CH of outcome
Course 1	3	22%	21%	19%	0.66 =(0.22 × 3)	0.63 =(0.21 × 3)	0.57 =(0.19 × 3)
Course 2	1	13%	11%	8%	0.13	0.11	0.08
Course 3	4	6%	4%	2%	0.24	0.16	0.08
Course 4	3	8%	7%	7%	0.24	0.21	0.21
Sum	11	-	-	-	1.27	1.11	0.94
Percentage	100%	-	-	-	Target = 1.27/11 =11.54%	Tool = 1.11/11 =10.09%	Score = 0.94/11 =8.54%

**Fig. 2:**
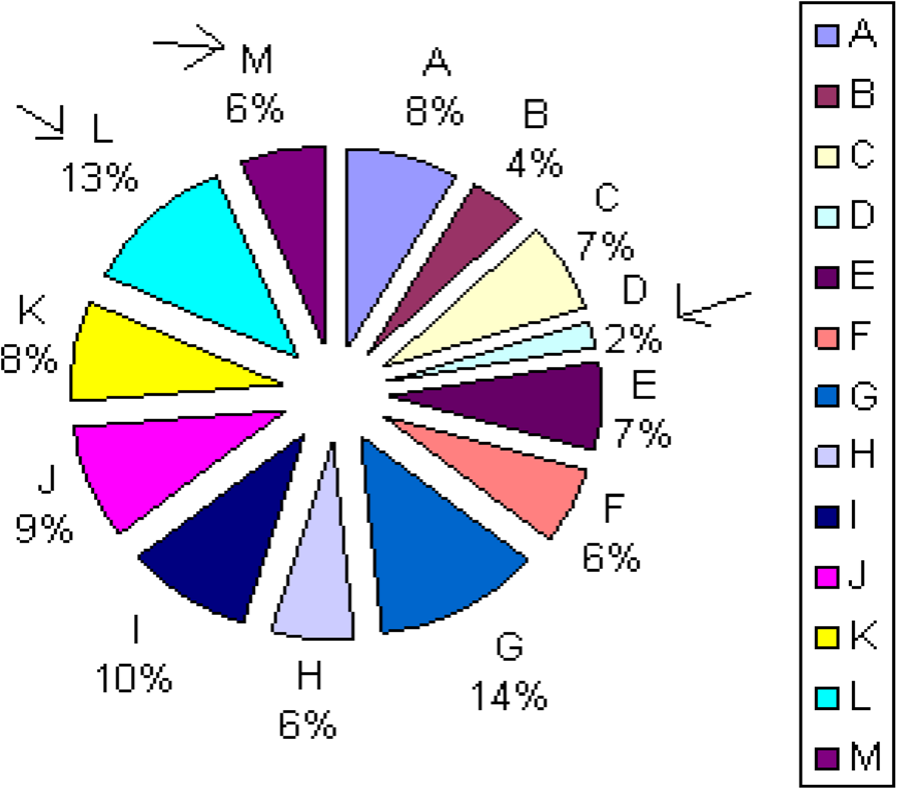
Target percentage distribution over all technical and medical outcomes in a BME curriculum (example). SOsM are represented by the sectors indicated by *arrows*. Every colored sector (*A-M*) represents an outcome targeted by the curriculum (10 technical and 3 medical outcomes). The sectors indicated by *arrows* (*D, L* and *M*) represent the medical outcomes SOsM. The percentage value and area of every sector indicate the percentage of credit hours targeting its related outcome in the BME curriculum, given the overall curriculum represents 100%

Figure 2 presents an example of *Target* distribution over technical and medical outcomes in BME curriculum calculated by Department Assessment Program. Every colored sector (A-M) represents an outcome addressed by the curriculum (10 technical and 3 medical outcomes). The sectors indicated by arrows (D, L and M) represent the medical outcomes SOsM: 1, 2 and 3, respectively. The percentage value and area of every sector indicate the percentage of credit hours- in the BME curriculum- addressing its related outcome, given the summation of all curriculum credit hours represents 100%. Note that the obtained distribution should be consistent with the curriculum *Target* values pre-determined by the program constituents at the beginning of the assessment cycle.

##### The quantitative result of the first assessment tool (course/curriculum assessment)

**Fig. 3:**
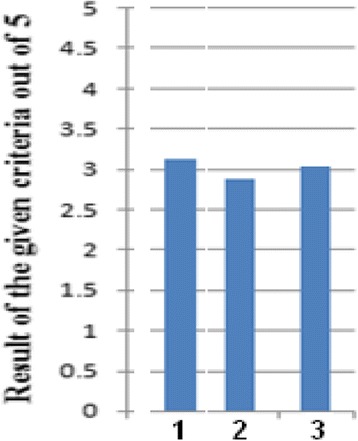
Course/curriculum assessment tool values (*Score*/*Tool*) for the medical outcomes 1–3 in a BME curriculum (example)

Afterwards, it is essential to conduct student performance assessment by calculating curriculum (*Score*/*Tool*) values transferred to a scale 1–5, i.e. when *Score* and *Tool* are equal, the result is 5. The obtained value is considered as the result of the first assessment tool (course/curriculum assessment). Figure 3 illustrates an example.

##### The quantitative result of the second assessment tool (curriculum assessment by students)

The curriculum assessment by students for every outcome can be obtained by: First, calculation of:

$\left(T \arg et\%\right)\times \left(\frac{CAS}{CAF}\right)$ for every course. Second, inserting the obtained values for all courses in DAP as ‘CAS2’ column. Third, inserting the *Target* values for every course in DAP. Fourth, calculation of *Target* and CAS2 values in terms of credit hours (the same procedure explained in Table 12 for *Target* and *Score*) for every outcome. Fifth, calculation of:

1 $$ \frac{\sum T \arg et{}CH}{\sum CH} $$

and

2 $$ \frac{\sum {CAS}_2{}CH}{\sum CH} $$

Finally, calculation of [(2)/(1)] and transforming the value to scale 1–5 i.e. the value is 5 when (1) and (2) are equal. We will not introduce a numerical example since the procedure is already discussed in Table 12. The value of [(2)/(1)] (scale 1–5) is considered as the result for this assessment tool (Course assessment by student).

#### Exit exam (3rd assessment tool)

The Exit exam given to graduating BME seniors is a comprehensive exam of approximately 60-min duration. The highest mark is 100. The exam questions were prepared on subjects related to BME courses. All exam questions were mapped to SOsM. ‘*Tool*’ values are consistent with curriculum pre-determined ‘*Target*’ values. *Tool* and *Score* values were calculated by the same procedure as implemented in final exam assessment tables presented above (Tables 4, 5, 6, 7, 8, 9, 10 and 11). The students performance, for every outcome, is calculated by (*Score*/*Tool*) transformed to the scale 1–5 e.g. the value is 5 when *Tool* and *Score* are equal. The (*Score*/*Tool*) value is considered as the result for this assessment tool (exit exam). An example question is:

Q) According to the Frank-Starling mechanism of the heart,

A) The left ventricle ejects a larger volume of blood with each systole than the right ventricle.
B) The intrinsic rate of the heart’s pacemaker is 100 beats/min.
C) Cardiac output increases with increased heart rate.
D) Stroke volume increases with increased venous return.

This question addresses outcome 2. The percentages for the outcomes 1–3 in the question are 0%, 100% and 0%, respectively. If the maximum mark of the question is, for example, 2 points then its contributions to the overall exit exam *Tool* values are 0, 2 and 0, respectively.

#### Capstone course (4th assessment tool)

The graduation project, in which a team of students work, represents the capstone design experience of the student. The evaluation of the graduation projects is done based on the information collected from the advisor grading as well as the final report and presentation assessment by examining jury. The assessment in the present work is based on the activities that are mapped to SOsM. The used documents can be found in [2, 38]. The results of graduation project assessment are processed as in the final exam assessment tables presented above (Tables 4, 5, 6, 7, 8, 9, 10 and 11). Every achievement expected from the graduation project is processed like a question with a contribution to *Tool* values. The students performance- for every outcome- is calculated by (*Score*/*Tool*) transformed to the scale 1–5 e.g. the value is 5 when *Tool* and *Score* are equal. An example of investigated achievement in the grading sheet is: safety, medical impact of the work & new medical trends. This expected achievement item addresses the outcomes 2 and 3. The percentages of outcomes 1–3 in this item are 0%, 50% and 50%, respectively. If the maximum mark of the achievement is, for example, 5 points then its contributions to the overall capstone *Tool* values are 0, 2.5 and 2.5, respectively.

#### Exit survey (5th assessment tool)

The exit survey provides valuable information on the student outcomes from the graduating senior class as they are leaving the program. The purpose of the exit questionnaire used in the present work is to gather information from graduating students on the level of program achievement of outcomes 1–3 in a 5-point scale. The used survey can be found in [2, 38]. Every question addresses one outcome. An example is:

Q. I have participated in the following learning experience: Interacting with medical practitioners/students.

1- Never 2- At Least Once 3- Several Times 4- Occasionally 5- Regularly.

This question addresses the outcome 1.

The result value of an outcome is the average of all answers (5-point scale) to the questions about that outcome. The result value is then reported as the output of this assessment tool (exit survey).

#### Alumni survey (6th assessment tool)

Alumni survey, mapped to SOsM, was circulated and collected at the BME alumni gathering day. The used survey can be found in [2, 38]. Alumni survey provides valuable information on the student outcomes (5-point scale) from the graduated biomedical engineers after leaving the program and being involved in different BM careers. Surveys were then analyzed. Every question addresses one outcome. The result value of an outcome is the average of all answers to the questions about that outcome. The result value (out of 5) is then reported as the result of this assessment tool (alumni survey).

#### Employer survey (7th assessment tool)

The medical centers feedback has been sought because there is close cooperation between BME departments and the healthcare institutions. The used survey can be found in [2, 38]. Survey forms were circulated during the alumni gathering day. Employed alumni were asked to forward these forms to the manager in the medical centers. They were requested to complete and return the survey forms. The form indicates the corresponding level of performance (out of 5) of BME employed graduates for each of the listed 1–3 outcomes based on comparisons with graduates of other comparable academic institutions or with other personnel assigned to the similar jobs.

#### Training in healthcare centers (8th assessment tool)

The survey designed to be filled by the training supervisors in the healthcare centers test the achievements of outcomes 1–3. The used documents can be found in [2, 38]. The form indicates the corresponding level of performance of BME student trainee (out of 5) for each of the listed 1–3 outcomes based on comparisons with other trainees of other comparable academic institutions. Training supervisors in hospitals and medical institutions are a constituency well qualified to provide assessment of the academic programs as well as knowledge, skills and character of students.

#### Suggested frequency and timeline

The implementation of the assessment tools discussed above should follow a pre-determined schedule (Table 13). The results of all assessment tools should be ready at the end of every academic year for revision and discussion by focus groups, a curriculum committee and department council. Furthermore, the recommendation of the department council should then be discussed with the advisory board and program constituents.

**Table 13:** Suggested schedule of assessment data collection

No.	Assessment tool	Analysis report frequency
1	Alumni Survey	Bi-Annually
2	Employer Survey	Bi-Annually
3	training Survey	Every Semester
4	Student Exit Survey	Every Semester Three weeks before the final exams of each semester
5	Instructor Assessment of Course	Every Semester End of a semester
6	Student Assessment of Course	Every Semester End of a semester
7	Capstone Assessment	Every Semester End of a semester
8	Exit Exam	Every Semester two weeks before the final exams of each semester

#### Calculation of weighted average

Every assessment tool results in a value within the range (1–5) for every student outcome related to biomedicine (SOsM). The weighted average is then calculated for every outcome (all assessment tools). The accurate weight of every assessment tool has been studied and investigated. The chosen weights (percentages) for curriculum, employer survey, training in healthcare centers, capstone course, exit survey, alumni survey, exit exam and course assessment by student are 30, 25, 10, 10, 10, 5, 5 and 5, respectively.

### Targeted evaluation/improvement of curriculum medical content in light of quantitative assessment results

**Fig. 4:**
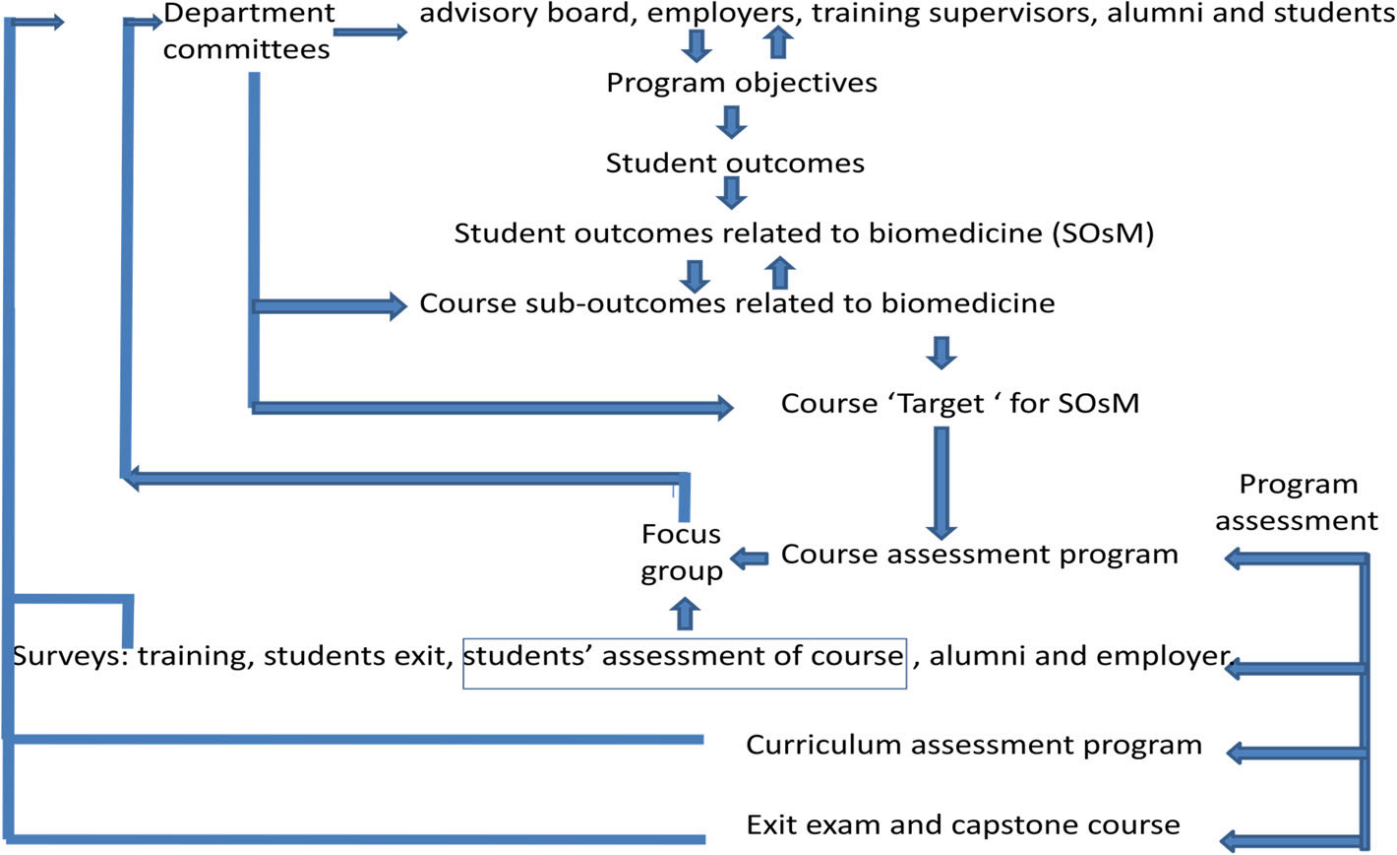
Continuous improvement cycle

The previous sections clearly showed a BME program has the 1–3 outcomes with varying values. Overall assessment indicates whether the weighted average of BME SOsM is a high, moderate or low success criterion. The improvement needed for the program is accordingly decided and specific actions should be taken in the curriculum to fill the gap. If the BME program achieves its targets in these key SOsM, this indicates that the BME curriculum is doing well in preparing the graduates from medical content point of view. Otherwise, the biomedical engineering curriculum committee is responsible for integrating changes into the curriculum. The changes are discussed and approved by the department council and advisory board. However, in the beginning of the continuous improvement process, the recommended changes -at the curriculum level- based on every assessment cycle are only adopted without application. After a number of assessment cycles, the changes are applied to the curriculum. Then, a new cycle of assessment begins. Only simple changes at course level (e.g. adding more examples to improve *Tool*) can be implemented immediately after an assessment cycle. This is due to the facts that, first, averaging over several assessment cycles eliminates irregularities or “noise” of values (out-of-trend values), second, the duration of a BME bachelor program is 5 years; the changes should not therefore confuse the students already following a certain study plan. Figure 4 presents the detailed framework of the process established. The process main elements are explained in the next sub-sections.

#### Review of program educational objectives and outcomes

Reviewing the list of educational objectives and student outcomes is part of the continuous improvement cycle. The departmental council reviews and approves the educational objectives and student outcomes. It compares their content with the content of the university, faculty, and department vision and mission. The departmental council also studies the evaluation of the educational objectives and student outcomes made by the department’s constituents. Extensive meetings overseen by the departmental committees involve relevant parties including the student body, public healthcare institutions, private sector representatives, department alumni, and potential employers. In the process, comprehensive surveys and questionnaires are distributed and analyzed. Note that the courses sub-outcomes and their *Target* values are modified accordingly. The courses are then delivered and assessed.

#### Improvements at course level

A major step in the improvements starts at the course level. Course level assessment is the responsibility of the individual faculty teaching the specific course. At the end of each course –normally after the end of the semester- the faculty member provides a course assessment form or a course report that evaluates the course and addresses issues that need attention in the next time the course is taught. The assessment is done through measuring the achievements of stated outcomes based on the course assessment program and the course assessment by student discussed in previous sections. Focus groups that are usually formed, based on the area of expertise, review assessment results for the courses and make recommendations for improving the course e.g. improving the organization of the course. These proposed changes are submitted to the curriculum committee for subsequent approval by the department council. The information requested by the focus group to improve the process is the summary of faculty observations in the current semester as well as the new measures implemented at the beginning of the semester considering focus group earlier recommendations. Table 14 illustrates an example of recommendations at the course level in light of the results.

**Table 14:** Example of recommendations by the focus group at the course level

Problem	Recommendation
(Target – Tool) > (N* × Target)	Assessment activities should be improved and diversified to cover all outcomes with correct/appropriate percentages.
(Tool -Score) > (N* × Tool) or (Score/Tool) < (M*)	The course should include more useful examples and delivery methods (related to the outcome) to improve learning. The course should also respect a gradual increase of sophistication.
Number of students passing more than (M* × Tool) are less than (P* × total number of students).	The course should include more useful examples and delivery methods (related to the outcome) to improve learning. The course should also respect a gradual increase of sophistication.
(CAF– CAS) > (T * × CAF)	- If problems 2 and 3 do not exist: Motivate students. - If problems 2 and 3 exist: The course should include more useful examples and delivery methods (related to the outcome) to improve learning.

*N, M, P and T are percentages set by the focus group, faculty member and department committee. Suggested: *N* = 30%. M = 60%, *P* = 60% and *T* = 30%

The final course documents (syllabus, sample of assessment activities, course final report presented by the instructor and the focus group…etc.) are placed in the course portfolio in case a new faculty member will teach the course. The course portfolio should be updated every semester.

#### Improvements at curriculum level

The second level of improvement lies in the curriculum development, which requires a comprehensive look at the courses taught, how they complement each other, and finally how they achieve the student outcomes. This in itself demands the active involvement of all program constituencies. The need for revising the BME curriculum stems from the informed/systematic analysis of assessment results, and input/feedback from the BME constituencies especially the advisory board.

All assessment-related feedback is collected and analyzed by the departmental committee. A report is generated for the assessment work. Evaluations regarding the level of achievement and recommendations for improvement are discussed in the departmental council and appropriate actions are taken (Table 15). A summary of findings from the report are discussed with constituencies including the advisory board, faculty, university administration …etc.

**Table 15:** Example of recommendations at the curriculum level

Problem	Recommendation
(curriculum Target values calculated by DAP –curriculum Target values determined a priori* by department) > (N** × curriculum Target values determined a priori* by department).	This means that the curriculum does not reflect perfectly the requirements of the program set by the department. Target values of every course should be revised.
(curriculum Target values calculated by DAP –curriculum Target values determined a posteriori** by department) > (N× curriculum Target values a posteriori*** determined by department)	This means that the constituents recommend modification of Target values in order to adapt to local and global market/research circumstances. Target values of every course should be revised. This case is mainly encountered in long assessment cycles.
Curriculum (Score/Tool) < 60% (or 3/5).	If all course-level improvements did not solve the problem then the structure and the sequence of curriculum should be revised. For example, additional pre-requisite or co-requisite courses should be inserted.
The weighted average of the results, of all assessment tools, calculated for an outcome is less than (3/5).	If all course-level improvements (courses sub-outcomes, courses Target values, courses assessment activities…etc.) did not solve the problem then a deep revision of curriculum structure and sequence should be carried out.
The result of one of the assessment tools is < (3/5) for an outcome.	- If the weighted average of all assessment tools is higher than (3/5) and the results of the high- weight assessment tools (most important) are satisfactory then no or only simple actions are implemented e.g. adding more activities related to the outcome in the courses. - If the weighted average of all assessment tools is lower than (3/5) then deep revisions should be carried out at course and curriculum levels.
(Target-Tool) > (N* × Target)	A revisiting of courses should be carried in order to achieve a curriculum total Tool value higher than threshold.
Change of outcome significance based on outcomes review by program constituents.	This means that the constituents recommend modifying the outcome translation into the curriculum in order to adapt to local/global market/research circumstances. This will induce a change in many courses sub-outcomes in order to make them complement each other and achieve the outcome new definition.
Addition or removal of one of the PEOs or SOsM based on outcomes review by program constituents.	This means that the constituents recommend modifying the outcomes list to adapt to local/global market/research circumstances. This will induce a change in curriculum. This will induce a change in many courses sub-outcomes in order to make them complement each other and achieve the new outcome.

A priori*: determined in the beginning of the current academic year/assessment cycle. N** is a percentage set by the focus group, faculty member and department committee. Suggested: *N* = 30%. A posteriori***: determined in the beginning of the following academic year/assessment cycle based on the review process by program constituents

### Implementation

The improvements have been applied to the curriculum of the BME department at JUST University based on the quantitative results, as example, in order to better explain and evaluate the process of filling in the gaps of a BME curriculum. All improvements have been mapped to the outcomes 1–3. The CAS assessment tool is applied to all 3rd, 4th and 5th grade students (240 students). The capstone, exit exam and exit survey are applied to the 5th grade students (51 students). The training supervisor, alumni and employer surveys are applied to 68 supervisors, 54 graduates (who graduated not more than 3 years earlier) and 32 employers, respectively.

## Results

### Identification of student outcomes related to biomedicine (SOsM)

The identified medical/technical outcomes and the procedure/criteria of identification are explained in the first section. The following section summarizes the quantitative data gathered and used to assess the quality of achievement of the outcomes 1–3.

### Separate quantitative assessment of every medical outcome SOsM by multiple quantitative assessment formats

The BME program performance in achieving SOsM (all assessment tools) is presented in Tables 16 and 17. The results in Table 16 are the obtained values before the application of weights. The results in Table 17 are the weighted values. As mentioned earlier, the chosen percentages (weights used in weighted average) for curriculum, employer survey, training in healthcare centers, capstone course, exit survey, alumni survey, exit exam and course assessment by student are 30, 25, 10, 10, 10, 5, 5 and 5, respectively.

**Table 16:** The results before applying the weights to the assessment tools

Outcome	Curriculum	Employer	Training	Capstone	Exit survey	Alumni	Assessment by students	Exit exam
1	3.1	3	2.70	5	3.65	3.16	2.66	2.02
2	2.9	3	4	4	3.46	3.26	2.64	0.95
3	3.3	3.5	4	4	3.63	3.3	2.32	1.11

**Table 17:** The results of averaging after applying weights to the assessment tools

Outcome	Curriculum	Employer	Training	Capstone	Exit survey	Alumni	Assessment by students	Exit exam	Sum
1	0.930	0.750	0.270	0.500	0.365	0.158	0.133	0.101	3.20
2	0.870	0.750	0.400	0.400	0.346	0.163	0.132	0.0475	3.10
3	0.990	0.875	0.400	0.400	0.363	0.165	0.116	0.0555	3.36

The results clearly show that the overall assessment of the BME program to which the suggested methodology is applied has a high success average of BME medical outcomes 1–3 (<3, SI: needs only “suggested Improvement”). Results show therefore, that the achievement of BME SOsM is satisfactory. However, a play of decided relative weights of assessment tools can induce change in results. The above weights have been selected to give more significance to the important tools as well as to the unbiased values. For instance, subjective values can unfortunately be frequently collected from answers/evaluations performed by students [2]. Also, not all students take the exit exam seriously as it is not counted in their GPA.

**Fig. 5:**
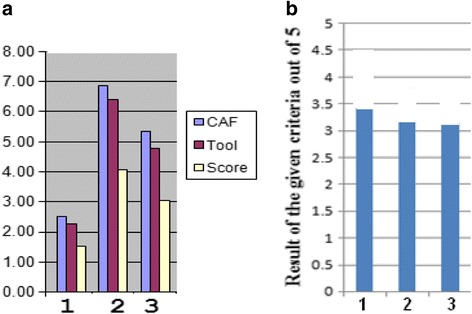
**a** The distribution of SOsM in terms of credit hours. **b** The corresponding values of (*Score*/*Tool*)

The *Target*, *Tool* and *Score* of SOsM in terms of credit hours, calculated by the department assessment program for 66 credit hours offered in the first semester to 3rd, 4th and 5th grades, are shown in Fig. 5a. *Target* and *Tool* values are fairly close i.e. (*Target* –*Tool*) is less than (0.3 × *Target*). Figure 5b illustrates the corresponding values of (*Score*/*Tool*) out of 5. All values are higher than 3.

**Fig. 6:**
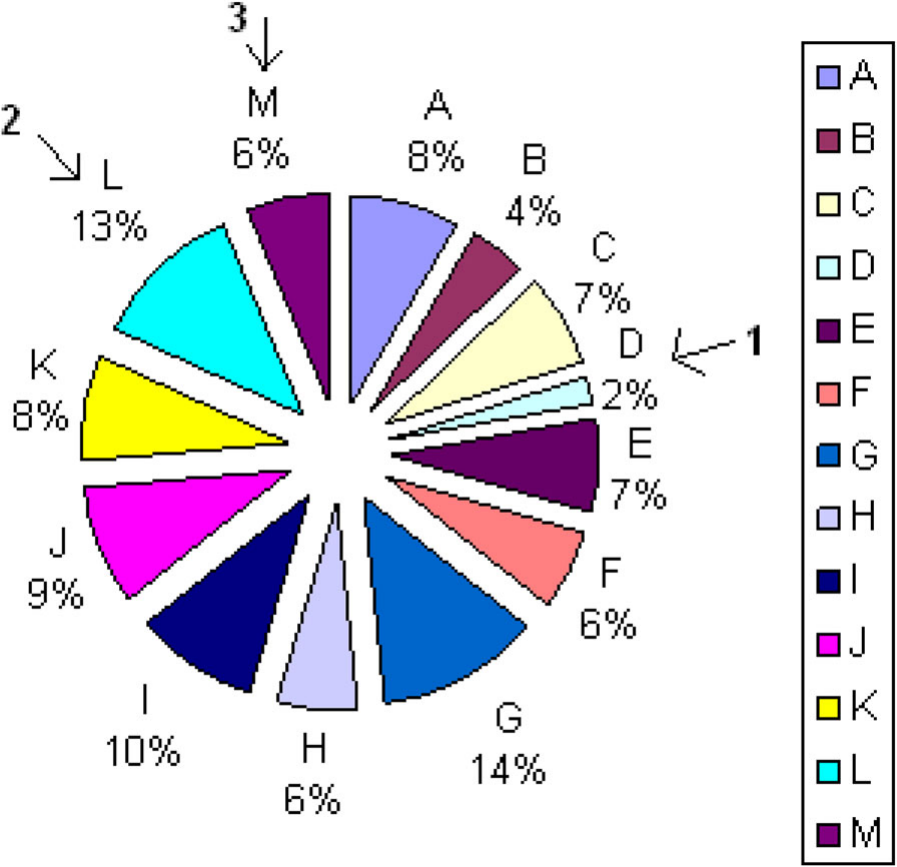
Distribution of BME credit hours according to the BME outcomes. SOsM are indicated by *arrows*. Every colored sector (*A-M*) represents an outcome targeted by the curriculum (10 technical and 3 medical outcomes). The sectors indicated by *arrows* (*D, L* and *M*) represent the medical outcomes SOsM. The percentage value and area of every sector indicate the percentage of credit hours targeting its related outcome in the BME curriculum, given the overall curriculum represents 100%

Figure 6 illustrates the distribution of curriculum *Target* values, calculated by the department assessment program for all credit hours offered during the assessment year to 3rd, 4th and 5th grades. SOsM are indicated by arrows. The obtained numbers are, in general, consistent with the values pre-determined by the department.

**Fig. 7:**
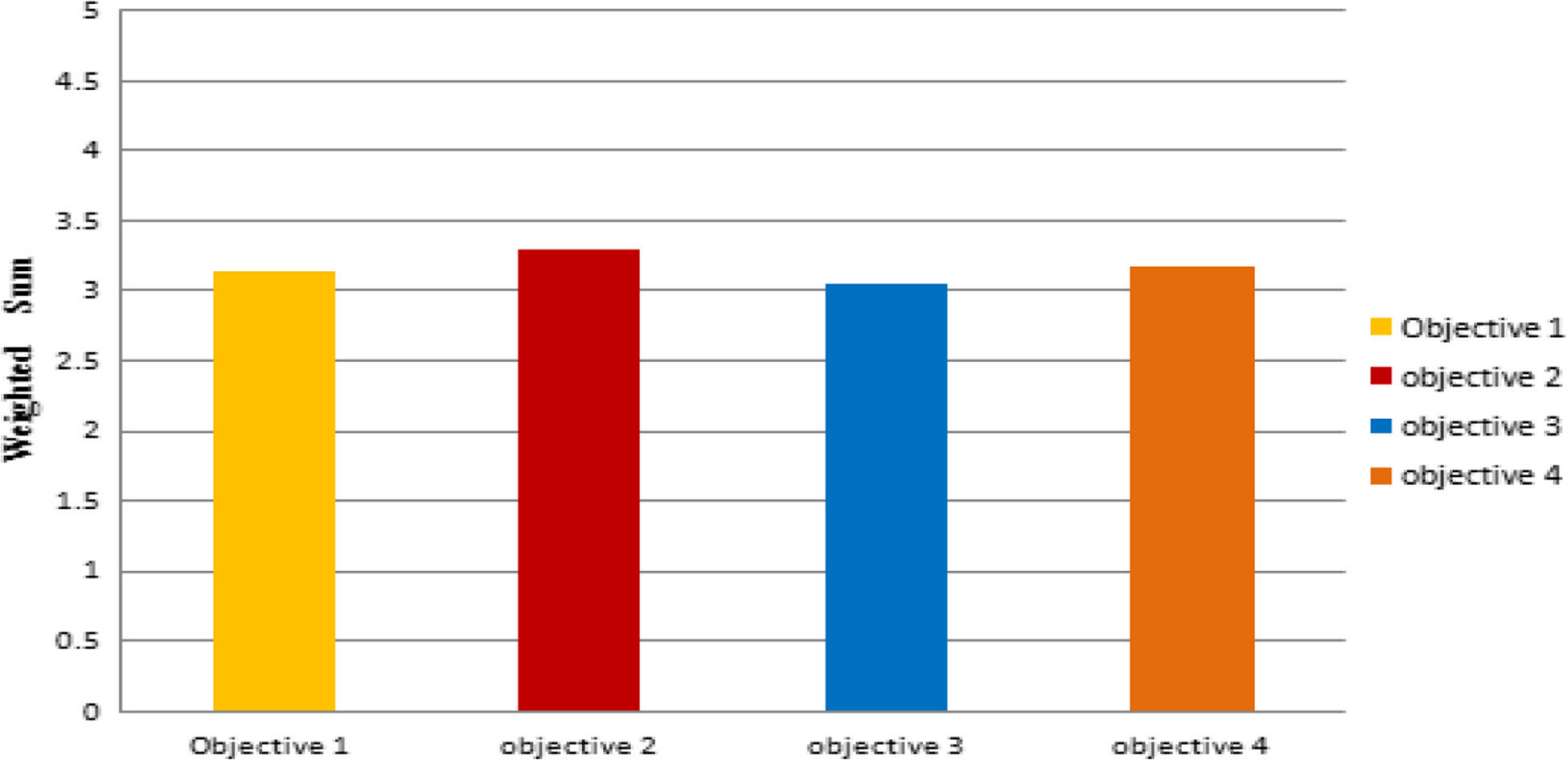
The degree of achievement of program educational objectives

Figure 7 illustrates the degree of achievement of program educational objectives. All obtained values are higher than 3. Since every PEO is a combination of a number of student outcomes (technical and medical), as discussed earlier, the calculated value is the average of the weighted averages of related outcomes. The high achievement of SOs leads to high achievement of PEOs.

### Targeted evaluation/improvement of curriculum medical content in light of quantitative assessment results

A few adjustments have been made for the content of some courses in the Biomedical Engineering Program. For instance, the physiology course has been changed to ‘Physio-anatomy’ to enforce living system knowledge. The main reason is the permanently low *Score* values related to the outcome 2, in the courses that necessitate anatomy knowledge (e.g. Biomechanics), despite all efforts made, at the course level, by the faculty members and focus groups (e.g. presenting introductory 3D videos that demonstrate anatomical concepts and asking the student to write a report). This is translated by the value 2.9 in Table 16. Table 18 illustrates the result of the course assessment program applied to the Biomechanics course. The program shows a warning message in the row of outcome 2 due to low *Score* (i.e. *Tool*-*Score* > 0.3*Tool) although the *Target* value indicates a high focus on the outcome 2 and although the *Tool* value indicates a very good set of assessment activities designed by the instructor (compared to the *Target* value). Subsequently, the focus group recommended modifying the sub-outcomes of physiology course in order to include anatomy and to create a pertinent pre-requisite course.

**Table 18:**
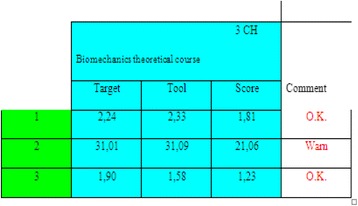
The results of course assessment program applied to the biomechanics theoretical course

The program shows a warning message in the row of outcome 2 due to low Score (*Tool* - *Score* > 0.3*Tool)

**Fig. 8:**
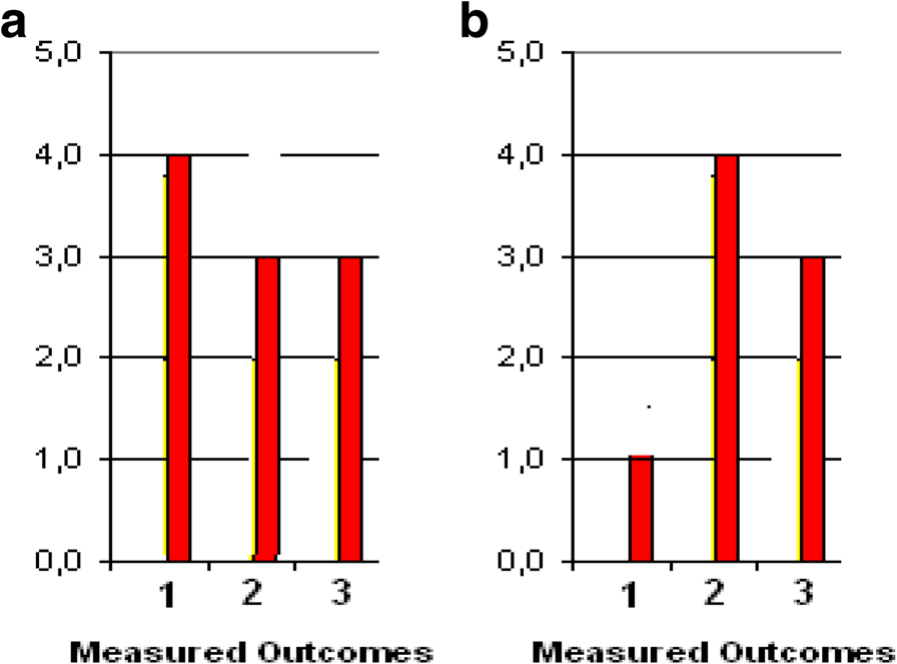
**a** CAF values (scale 1–5) for SOsM in the course “BME seminar”. **b** CAF values (scale 1–5) for SOsM in the course “Nanomedicine”

The second level of improvement lies in the curriculum development, which requires a comprehensive look at the courses taught, how they complement each other, and finally how they achieve SOsM. Based on the department recommendations, the 2% *Target* value (Figure 6) for outcome 1 should be slightly improved. Note that although the weighted average for outcome 1 is higher than 3, the assessment value given by the training supervisors is 2.7 (important assessment tool) (Table 16). Consequently, new courses e.g. ‘Introduction to biomedical engineering’ and ‘BME seminar’ have been added to introduce students to multidisciplinary team/group work where they encounter situations with medical practitioners and students (outcome1) as illustrated in Fig. 8a. Furthermore, as far as the medical contemporary and cutting edge issues in BME are concerned, the *Target* values for outcomes 2 and 3, in the curriculum, should be adjusted accordingly. Thus, the program constituents recommended adding a number of core BME classes to tackle the evolution in biomedicine e.g. courses related to cell and molecules. A number of elective courses were also introduced in the curriculum e. g. Nanomedicine as illustrated in Fig. 8b.

The results also show a low value of *Tool* (compared to *Target*) for the SOsM in the BME courses taught by the pure technology departments and faculties at the university (e.g. Control systems). This is due to the fact that the courses lack activities that imply examples on applications of medical aspects. The improvement is achieved through incorporating those classes into the BME department (e.g. “Control and Communication in the Nervous System”, “Laboratory of Physiological Control”…etc.). This will add a medical component. Table 19 illustrates a comparison between the *Tool* values of ‘Laboratory of Control Systems’ and ‘Laboratory of Physiological Control’ based on the activities indicated in the class work manuals of both courses.

**Table 19:**
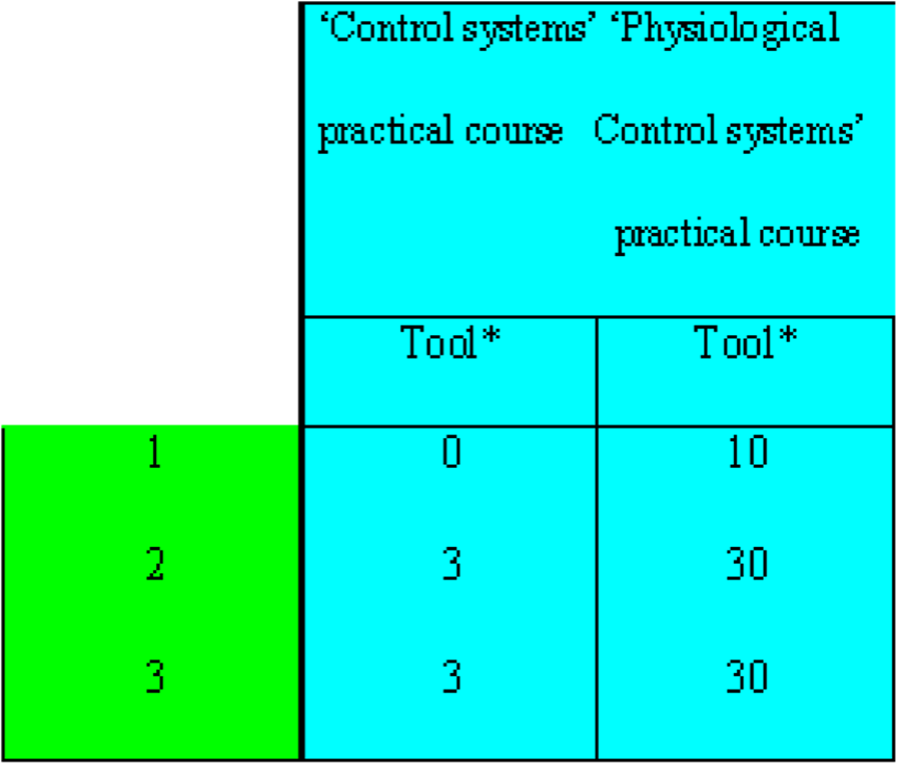
The comparison between the *Tool* values of ‘Laboratory of Control Systems’ and ‘Laboratory of Physiological Control’

Tool*: based on the activities indicated in the class work manual of the course

Table 20 shows example of mapping between a few selected courses and the BME SOsM.

**Table 20:** Example of mapping of a few selected BME courses, in the new curriculum, to BME SOsM

Course title	1	2	3
General Biology I		×	×
General Biology Lab		×	×
Physioanatomy		×	×
Physiological Fluid		×	
Physioanatomy Lab	×	×	×
Biochemistry		×	
Physiological modeling lab	×	×	×
Biomedical Transport phenomenon	×	×	×
Nanomedicine	×	×	×
Artificial Organs		×	×
Control and Communication in the Nervous System		×	×

### Implementation

Table 21 illustrates the preliminary results of progress of the assessment indicators from the old curriculum to the new curriculum. The values are clearly improved. As the improvements have not been applied for a long time, we do not include the progress of the values related to the alumni, employer, exit and training surveys as well as the exit exam and capstone. Also, the comparison includes only the courses of 3rd and 4th grades.

**Table 21:** Progress of curriculum assessment

Outcome	[(Target-Tool)/Target]%		(Score/Tool) in scale 1–5		Assessment by students	
	Old	New	Old	New	Old	New
1	8.12%	6.32%	3.1	3.3	2.66	3.01
2	14.78%	7.14%	2.9	3.5	2.64	2.97
3	13.11%	6.55%	3.3	3.5	2.32	2.76

## Discussion

### Identification of student outcomes related to biomedicine (SOsM)

The present work serves as a guiding illustration to demonstrate a model of analysis since every BME curriculum has its own special elements and characteristics based on the institution’s goals. The proposed procedure can be generalized and hence used by any BME department. However, intended outcomes are subject to variability among universities. Consequently, SOsM stated above can be modified to adapt the analysis to every department mission, vision and objectives. Success criteria can also vary from one department to another. Departments focusing on biomaterials, artificial organs or neural aspects can attribute higher values to success criteria than those focusing on more technological issues.

The followed outcome structure might appear different than the existing international medical outcome frameworks in the world; e.g. CanMEDS, ACGME outcomes, Scottish Doctor Outcomes. This is because the international medical learning outcome frameworks in medical education have at least five or more learning outcomes while the followed structure in the present work has three outcomes. For example the CanMEDS roles (physician competencies) are: Medical expert (the integrating role), communicator, collaborator, leader, health advocate, scholar and professional [41]. The ACGME outcomes focus on patient care, medical knowledge, practice-based learning and improvement, interpersonal and communication skills, professionalism, and systems-based practice [42]. Scottish Doctor Outcomes focus on learning outcomes for clinical skills, practical procedures, patient investigation, patient management, communication, health promotion and disease prevention, medical informatics, social and clinical sciences and underlying principles, ethical understanding and legal responsibilities, decision making skills, clinical reasoning and judgment, role of the doctor within the health service, and personal development [43]. However, if we examine carefully the applied outcome structure of the present work, we will find out that the dimensions (D1 to D5) and PEOs (PEO1 to PEO4) are almost similar to other internationally defined outcomes. Those dimensions and PEOs encompass not only the three medical skills and attributes but also the ABET technical outcomes (13 outcomes in overall). All outcomes complement each others to achieve the overall expected goals of the multidisciplinary BME curriculum, dimensions and PEOs. In addition, it is worthy to note the fact that the BME curriculum has a special-case medical content that is not supposed to target all the skills and the attributes (outcomes) expected from a medical student/doctor/physician in medicine; it is supposed to target the outcomes helping the BME students succeed in the medical field as future medical engineers. For example, a medical doctor/physician should be a good medical problem solver in medical diagnosis issues while a medical engineer should be a good technical problem solver in issues of application of engineering tools to medical diagnosis. The outcome of problem solving is therefore ‘dominated’ by the technical side and hence considered as a technical outcome. Other examples are the objectives of leadership and decision making ‘dominated’ by the technical side. Overall, the expected abilities will certainly not be underrepresented by the suggested structure (technical and medical outcomes altogether). Furthermore, the present work can be flexibly adapted to any other studied BME curriculum by interchanging a few outcomes between the lists of medical and technical outcomes based on the extent of ‘domination’ as perceived by the curriculum objectives. However, at the end of process, the overall adapted structure should be verified in order to represent perfectly the aimed dimensions and PEOs.

### Separate quantitative assessment of every medical outcome SOsM by multiple quantitative assessment formats

Many BM engineers are not confident of their medical knowledge. The difficulty is mainly produced by the weak medical content in the curriculum [34]. The proposed work is a promising approach that can solve the problem. The suggested assessment tools are useful for evaluation of BME students’ expected capacity in the medical field. They are also helpful in cases of curricula addressing a mix of populations with medical and engineering backgrounds or interests.

The presented quantitative approach has many advantages compared to the qualitative methods: validity (measures exactly the outcomes), generalizability, reliability (repeatable results) and objectivity (low bias) [27]. In the presented work, the methodology relies on more than one quantitative assessment tool to ensure high accuracy. Quantitative tools are very important for the evaluation of the multidisciplinary BME curriculum because the level considered as the ‘minimum expected level’ for every medical outcome is a very sensitive parameter. For example, if a BME department offers a curriculum with two tracks: (1) biomedical instrumentation and (2) biomaterials and biomechanics then the students in both tracks are expected to demonstrate adequate knowledge of physiology. However, the minimum expected levels of “adequacy” are different. The difference can be simply detected by quantitative approaches (e.g. *Target*, *Score* …etc.). Conversely, it is not easy to discover the difference by qualitative approach. In addition, the levels of “adequacy” of physiology knowledge, in both tracks, should not overcome the level of “adequacy” of the other medical or technical knowledge. However, the permitted extents of flexibility/tolerance in the tracks are dissimilar. It would be difficult to measure the tolerance by qualitative tools.

In [28], the collaborators analyzed a number of qualitative surveys that helped them plan the improvement of the medical content in the BME department through building a relationship with medical schools. However, they did not measure the extent of improvement. The approach did not permit to quantify the precise difference between the curricula before and after. In [29], the medical content has been improved by the problem-based integration of medical applications in engineering courses. Nevertheless, the study did not approach the effect on the whole curriculum structure and sequence. On the other hand, the research in [3, 30] which focused on the details of curriculum structure and development did not measure the impact on the students’ outcomes and performance. It was rather interested in a program that can attract medical doctors and engineers, at the same time, without evaluating the level of medical knowledge for an engineer in comparison with another BME curriculum. In [31], the BME curriculum is developed in consistency with the department’s clear-cut mission to realize courses with specific characteristics and rich in medical content. However, the assessment was only conducted by the survey of students’ satisfaction. The authors in [4] discussed the management, timeline and structure of a biomedical engineering based program organized for medical students. Yet, the program goals and expected progress are the only items investigated. In [32], a spiral approach has been conducted by revisiting of topics with increasing levels of difficulty/sophistication to enhance students’ competency in medical and engineering fields. Conversely, the students’ competency is measured with simple surveys and interviews. Finally, in [33], the authors discussed the improvement of medical content of the BME curriculum by following the models in high standard international schools. The improvement is mainly carried out by adding appropriate elective courses. However, the interaction between the curriculum content and the level of student skills is not approached. In all of the previous mentioned studies, the medical outcomes are not clearly/separately identified or they are not precisely assessed; the assessments depend mainly on one type of survey/questionnaire or on students’ overall grades. The present work shows that the clear identification of expected outcomes and the accurate assessment of achievement are strong tools of curriculum medical content evaluation/improvement.

### Targeted evaluation/improvement of curriculum medical content in light of quantitative assessment results

In general, the present work is “user-friendly” so that it can be implemented smoothly in a continuous manner. However, the improvement of the courses can be tricky if the instructor does not map activities properly, does not give sufficient activities to cover the outcomes, or if he carries out the evaluation in a very general way. Errors in the improvement at the course level can induce cumulative errors in the curriculum level, which can affect the whole improvement procedure. Furthermore, as showed in the ‘Results’ section, the individual assessments by every tool should not be neglected although the overall weighted average is calculated. The evaluation made by every assessment tool can give valuable information, especially the highly-weighted tools. For example, outcome 2 was attributed a value >3 in overall assessment and a value slightly less than 3 in curriculum assessment. This was not ignored as shown in the previous section. In addition, outcome 1 was attributed a value >3 in overall assessment and a value less than 3 by training supervisors. This was also taken into account.

## Conclusion

The degree of achievement of SOsM is an essential indicator for medical content in the BME program. It has been estimated, in the present work, by a number of quantitative assessment tools. The tools have been applied and analyzed to uncover the weakness in the curriculum. Subsequent improvements have been conducted to fill in the gaps.

The suggested assessment tools can be generalized and extended to any other BME department. Robust improvement of medical content in the BME curriculum can subsequently be achieved.

Future work in next cycles of assessment will hit upon the new curriculum to conduct further continuous improvements and to evaluate students’ performance compared to previous cycles. In addition, more quantitative ‘zooming’ will be applied to the SOsM at the level of medical sub-outcomes in order to increase the accuracy of assessment.

## Abbreviations

ABET: Accreditation Board for Engineering and Technology
BME: Biomedical Engineering
CAF: Course assessment by faculty
CAP: Course assessment program
CAS: Course assessment by student
DAP: Department Assessment Program
EAC: Engineering Accreditation Commission
GP: Graduation Project
JUST: Jordan University of Sciences and Technology
MI: Major improvement
NI: Needs improvement
PEOs: Program Educational Objectives
SI: Suggested improvement
SOs: Students outcomes
SOsM: Students Outcomes related to Medicine and Biology/or to Biomedicine/or Student Medical Outcomes
